# A natural approach to combating antibiotic-resistant pathogens in livestock: *Hibiscus sabdariffa*-derived hibiscus acid as a promising solution

**DOI:** 10.17221/105/2023-VETMED

**Published:** 2024-06-27

**Authors:** Esmeralda Rangel-Vargas, Carlos Alberto Gomez-Aldapa, Reyna Nallely Falfan-Cortes, Fabiola Araceli Guzman-Ortiz, Javier Castro-Rosas

**Affiliations:** ^1^Academic Area of Chemistry, Institute of Basic Sciences and Engineering, City of Knowledge, Autonomous University of the State of Hidalgo, Mineral de la Reforma, Mexico; ^2^Professor of the National Council of Science, Humanities and Technology, Crédito Constructor, Benito Juárez, Mexico City, CDMX

**Keywords:** plant antimicrobial agents, *Salmonella* Typhimurium, Shiga-toxin-producing *Escherichia coli*, synergistic effect

## Abstract

We examined the antibacterial efficacy of streptomycin, hibiscus acid, and their combination against multidrug-resistant Shiga-toxin-producing *Escherichia coli* (STEC) and *Salmonella* Typhimurium in mice. We determined the minimum inhibitory concentration (MIC) and minimum bactericidal concentration (MBC) for streptomycin, hibiscus acid, and their combination against STEC and *Salmonella*. Fifteen sets of six mice in each set were utilised: six groups were orally exposed to 4 log_10_ colony forming units (CFUs) of *S. *Typhimurium and another six to STEC, and three acted as the controls. Six hours post-inoculation, specific groups of mice received either oral solutions containing hibiscus acid at 5 and 7 mg/ml; streptomycin at 50 and 450 μg/ml; hibiscus acid/streptomycin (5 mg/ml hibiscus acid and 50 μg/ml streptomycin); or isotonic saline. The study determined the MIC and MBC of 7 mg/ml of hibiscus acid; 300 and 450 μg/ml of streptomycin; and two concentrations of hibiscus/streptomycin (3 mg/ml / 20 μg/ml and 5 mg/ml / 50 μg/ml). Interestingly, the mice that were infected and subsequently treated with hibiscus acid at 7 mg/ml alone or in conjunction with streptomycin did not have either STEC or *Salmonella* in their faecal samples, and none of the mice died. In contrast, the untreated mice and those exclusively treated with streptomycin had the pathogens present in their stool, leading to the mortality of all the subjects.

The escalating crisis of antibiotic resistance is an imminent threat to both human and animal health worldwide (WHO 2023). Within the realm of veterinary medicine, the emergence and dissemination of antibiotic-resistant bacteria among livestock populations presents a significant challenge, with pathogens such as *Salmonella* and Shiga-toxin-producing *Escherichia coli* (STEC) at the forefront of concerns (Karch et al. 1999; Boore et al. 2015; WHO 2023). As we face the looming prospect of a post-antibiotic era, the need for innovative approaches to combat antibiotic-resistant pathogens in veterinary medicine has never been more pressing.

STEC and *Salmonella* have emerged as significant and concerning health hazards. These two microorganisms, classified as foodborne pathogens, pose a substantial risk to public health on a global scale. The prevalence of outbreaks attributed to these pathogens has garnered considerable attention, prompting intensified research and surveillance efforts to mitigate their impact (Karch et al. 1999; Boore et al. 2015).

STEC, a subset of *E. coli* bacteria, is characterised by its ability to induce haemorrhagic colitis, a condition marked by severe abdominal pain and bloody diarrhoea. This bacterium is of grave concern due to its propensity to cause outbreaks, with instances of contamination often traced back to the consumption of undercooked ground beef, unpasteurised dairy products, or contaminated produce. The virulence of STEC lies in its production of *Shiga*-toxins, which can lead to haemolytic-uremic syndrome (HUS), a condition characterised by renal failure and potential long-term health consequences (Beutin and Martin 2012).

Another notorious foodborne pathogen is *Sal-monella*, which comprises a diverse group of bacteria capable of causing gastroenteritis in humans and animals. The impact of *Salmonella* infections can range from mild gastrointestinal discomfort to severe dehydration. The sources of *Salmonella* contamination are multiple, including poultry, eggs, raw meat, and even fresh produce (CDC 2013).

The livestock industry, a key component of global food production, is critically impacted by the rise of antibiotic resistance. Conventional antibiotics, once effective in promoting animal health and ensuring food safety, are losing their potency due to the relentless adaptation of bacterial strains. The result is not only compromised animal welfare, but also a direct threat to human health due to the potential transmission of resistant pathogens through the food supply chain. *Salmonella* and STEC have become serious health threats as globally important foodborne pathogens causing numerous outbreaks (Chang et al. 2015).

This dire situation necessitates novel approaches to treating bacterial infections. In this context, the rich biodiversity of plant species has captured the attention of researchers as a potential source of natural antibacterial agents. Such agents hold the promise of yielding innovative compounds that could be used to control infections on a global scale. The medicinal potency of plants lies within their complex array of secondary metabolites, including alkaloids, flavonoids, terpenoids, and phenolic compounds (Cruz-Galvez et al. 2013; Ma et al. 2019).

*Hibiscus sabdariffa* is a species of subtropical plant that grows in countries such as Mexico, Sudan, India, and Thailand. Recently, Portillo-Torres et al. (2019), reported that hibiscus acid obtained from acetonic extract of *H. sabdariffa* calyces is one of the compounds responsible for the antibacterial activity of *H*. *sabdariffa*. Recently, the antibacterial effect of hibiscus acid on different microorganisms such as *Salmonella* serotypes (Portillo-Torres et al. 2019; Sedillo-Torres et al. 2022), *E. coli* (enteroinvasive, enteropathogenic, enterohemorrhagic and Shiga toxin-producing) (Portillo-Torres et al. 2019), *Streptococcus mutans*, *S. sanguinis*, *Capnocytophaga gingivalis*, and *Staphylococcus aureus* (Baena-Santillan et al. 2022), and *Pseudomonas aeruginosa* (Cortes-Lopez et al. 2021) has been reported. Although the complete mechanism of the effect of hibiscus acid on the bacterial cell is not well known, evidence suggests that hibiscus acid alters the bacterial membrane (Portillo-Torres et al. 2019; Baena-Santillan et al. 2022; Sedillo-Torres et al. 2022), inhibits the flagellar motility and cell invasion in *Salmonella enterica* (Sedillo-Torre et al. 2022), and has a strong interaction with the active site of the LasR protein (Cortes-Lopez et al. 2021). However, more studies are necessary to elucidate the complete mechanism of the effect of hibiscus acid on bacteria. In addition, Baena-Santillan et al. (2022) reported that hibiscus acid is not toxic. Other authors have also mentioned that hibiscus acid is not toxic (Zheoat et al. 2019; Sedillo-Torres et al. 2022).

Currently, there is no information on the possible antibacterial effects of hibiscus acid when administered in an animal model infected with pathogenic, antibiotic-resistant bacteria. In the literature, there is currently only one study available on the antimicrobial effect of hibiscus acid in a mouse abscess/necrosis model, hibiscus acid at sublethal concentrations (15 and 31.2 μg/ml) that affected infection establishment by *P. aeruginosa* and prevented damage and systemic spread (Cortes-Lopez et al. 2021).

We recently reported on a study on the antimicrobial effects of aqueous extract from calyces of *Hibiscus sabdariffa* in CD-1 mice infected with multidrug-resistant enterohaemorrhagic *E. coli* (EHEC) and *S. *Typhimurium (Portillo-Torres et al. 2022). In the reported study, the effect of the aqueous extract of *H.* *sabdariffa* calyces alone and at a concentration of 50 mg/ml was tested in CD-1 mice orally infected with EHEC or *S*. Typhimurium. EHEC and *S*. Typhimurium were absent in the faecal samples of the mice that received the aqueous extract on the 2^nd^ and 3^rd^ days after treatment. Additionally, these mice showed signs of recovery from the infection. Conversely, in the untreated mice or those treated solely with chloramphenicol, the pathogens persisted in their faeces throughout the study, leading to the mortality in some mice (Portillo-Torres et al. 2022).

The objective of this study is to determinate the minimum inhibitory concentrations (MICs) and minimum bactericidal concentrations (MBCs) for streptomycin, hibiscus acid, and their blend against multidrug-resistant STEC and *Salmonella*; and to evaluate the antibacterial effects of hibiscus acid when administered, alone or in combination with the antibiotic streptomycin, to mice infected with multidrug-resistant STEC and *S.* Typhimurium.

## MATERIAL AND METHODS

### Isolation of hibiscus acid

A kilogram batch of dehydrated calyces of *H.* *sabdariffa* cultivated in the state of Guerrero, Mexico, was used to obtain hibiscus acid from an acetonic extract as described by Portillo-Torres et al. (2019). Briefly, samples (100 g) of dehydrated calyces were placed in glass flasks and 900 ml of acetone were added. The flasks were hermetically sealed and stored at room temperature for 7 days with manual shaking for 1 min once a day. Afterwards, the liquid phase was filtered through filter paper. The filtered extracts were concentrated in a rotary evaporator. The acetone was completely removed from the rotary evaporated concentrate by placing it in an air recirculation oven at 45 °C for 24 hours. Two hundred and thirty grams (230 g) of dry acetone extract of *H. sabdariffa* calyces was packed with silica gel in a chromatographic column. Hexane was used as the mobile phase to separate the oils in the extract, and 600 ml fractions were recovered in glass flasks. All the chromatographic fractions obtained were rotary-evaporated to remove the solvents and concentrate the separated compounds. After discarding most of the oils from the extract, the solvent mixture hexane-ethyl acetate (9 : 1 v/v) was used as the mobile phase to remove all the residual oils. The mobile phase (8 : 2 v/v) passed through the packed column until some small crystals were observed in the rotary-evaporated fractions and it was then used at a ratio of 7 : 3 (v/v) to obtain well-defined crystals in the rotary-evaporated fractions. It was re-crystallised using 7 : 3 (v/v) acetone–ethyl acetate in a separatory funnel and then stored for 24 hours. Once the formation of crystals on the wall of the separation funnel was observed, the liquid was decanted, and the crystals were recovered. Finally, the residual acetone was removed in an air recirculation oven at 45 °C for 2 hours.

### Bacterial strains

Four multidrug-resistant bacteria strains were isolated from different foods. *S*. Typhimurium C12 and S70 (both resistant to 12 antibiotics) were isolated from coriander (Rangel-Vargas et al. 2016) and tomatoes (Gutierrez-Alcantara et al. 2016), respectively; and STEC CA1 (*Stx2* gene and resistant to 14 antibiotics) and BJ22 (*Stx2* and resistant to 12 antibiotics) were isolated from fresh cheeses made with unpasteurised milk (de la Rosa-Hernandez et al. 2018). All the bacteria were resistant to the same 11 antibiotics (amoxicillin–clavulanic acid, amikacin; ampicillin, colistin, erythromycin, gentamicin, kanamycin, neomycin, sulfisoxazole, trimethoprim-sulfamethoxazole and streptomycin) according to the protocol indicated by the Clinical and Laboratory Standards Institute (CLSI 2020). It is important to note that for all the studies, mutants resistant to rifampicin were used, which were obtained from the four multidrug-resistant STEC and *Salmonella* described above. Rifampicin-resistant mutant strains (+; Sigma-Aldrich, Ciudad de México, Mexico) of *S.* Typhimurium, and multidrug-resistant STEC strains resistant to other antibiotics were obtained according to the method described by Castro-Rosas and Fernandez-Escartin (2000). These mutant rifampicin-resistant strains were chosen specifically to ensure accurate tracking and analysis. To facilitate this monitoring, colony counts were conducted on agar plates containing rifampicin.

The use of the rifampicin-resistant strain served the dual purpose of incorporating rifampicin into the agar plates, thereby creating an environment where only the targeted bacteria could thrive, while simultaneously preventing the growth of other bacterial strains. It is worth noting that rifampicin is a restricted antibiotic, uncommonly utilised in both human and veterinary medicine. Consequently, naturally occurring bacterial resistance to rifampicin is minimal, further underscoring its suitability for this study’s experimental design. This approach enhances the reliability of the microbiological results.

### Inocula preparation

All four of the multidrug-resistant STEC^+^ and *S.* Typhimurium^+^ strains were inoculated in trypticase soy broth (TSB) and incubated at 35 °C for 18 hours. The cultures were washed twice in sterile isotonic saline solution (ISS; 0.85% NaCl) by centrifuging at 2 000 *g* for 20 min, and the pellets were resuspended in sterile peptone water at about 9 log_10_ CFU ml. An inoculum cocktail was prepared for each multidrug-resistant strain by mixing 1 ml of each washed suspension.

### Minimum inhibitory concentration and minimum bactericidal concentration

To determine of the minimum inhibitory concentration (MIC) and minimum bactericidal concentration (MBC), we used the macrodilution method using the inocula cocktail of* S*. Typhimurium^+^ or STEC^+^ at 1 × 10^5^ CFU/ml as described by Portillo-Torres et al. (2019). We applied some variations: we used trypticase soy broth (TSB) tubes containing hibiscus acid, streptomycin, and hibiscus acid/streptomycin mixtures at different concentrations. All the treatments were performed in triplicate.

### Fractional inhibitory concentration index (FICI)

The fractional inhibitory concentration index (FICI) is a numerical value used to assess the interaction between two or more antimicrobial agents when administered in combination. We estimated the FICI value between the hibiscus acid and streptomycin using the MIC values of each of the compounds on both pathogenic bacteria, using the following equation (Garvey et al. 2011):

$$\text{FICI}=\frac{\begin{array}{c} \text{MIC streptomycin}\\ \text{in combination} \end{array}}{\begin{array}{c} \text{MIC streptomycin}\\ \text{alone} \end{array}}+\frac{\begin{array}{c} \text{MIC hibiscus acid}\\ \text{in combination} \end{array}}{\begin{array}{c} \text{MIC hibiscus acid}\\ \text{alone} \end{array}}$$ (1)

The FICI results were interpreted as follows: < 0.5 indicates synergism; 0.5–1 indicates an additive effect; > 1–2 indicates indifference or no effect, and > 2 indicates antagonism.

### The antimicrobial effect in CD-1 mice infected with STEC or *S.* Typhimurium

We investigated the antimicrobial effect of hibiscus acid, streptomycin, and the hibiscus acid/streptomycin mixture in strain CD-1 mice infected with rifampicin-resistant mutant strains (+) of STEC or multidrug-resistant *S.* Typhimurium strains resistant to other antibiotics. The use of the CD-1 mice strain is justified based on several factors. CD-1 mice are a commonly used outbred strain in biomedical research due to their genetic heterogeneity, which closely mimics the genetic diversity found in human populations (Aldinger et al. 2009). This genetic diversity can influence the host response to infections and treatments, making CD-1 mice suitable for studying complex interactions between pathogens and potential therapeutics (Aldinger et al. 2009). In addition, CD-1 mice have been extensively employed in infectious disease research, including studies involving *Salmonella* (Ramachandran et al. 2017) and STEC (Mohawk and O’Brien 2011).

We conducted this study as described by Portillo-Torres et al. (2022). Briefly, an inocula cocktail (1 × 10^5^ CFU/ml) of STEC^+^ or *S.* Typhimurium^+^ was used. Ninety healthy male mice of the CD-1 strain of 8 weeks of age were used. The experimental protocol involving mice was analysed and approved by the University (UAEH) Ethics Committee for the Care and Use of Laboratory Animals. For inoculation into mice, MBCs against both STEC^+^ and *S.* Typhimurium^+^ cocktail were used. The concentrations of the used test solutions were therefore 7 mg/ml, 450 μg/ml, and 5 mg/ml / 50 μg/ml for hibiscus acid, streptomycin, and hibiscus acid/streptomycin, respectively. The 90 mice were divided into 15 groups of six mice each (groups I to XV). All the groups were maintained for 1 week of adaptation, providing them with standard food and water *ad libitum*. After this adaptation time, the mice were orally inoculated with an inocula cocktail of STEC^+^ or *S.* Typhimurium^+^. The mice were held firmly by the scruff of the neck in a vertical position and inoculated with the R^+^ pathogen in suspension, antibacterial solution, or saline solution, using an oesophageal cannula attached to a sterile needleless syringe. Mouse group I was not infected with the pathogenic strains, and no treatment was administered (blank, only an isotonic saline solution (ISS) was administered orally). Groups II and III were not infected with the pathogenic strains, but they were administered streptomycin and hibiscus acid, respectively (uninfected and treated controls). Groups IV, VI, VIII, X, XII and XIV were inoculated orally (0.1 ml) with approximately 1 × 10^4^ CFU of the *S*. Typhimurium^+^ cocktail. Groups V, VII, IX, XI, XIII and XV were inoculated orally (0.1 ml) with approximately 1 × 10^4^ CFU of the STEC^+^ cocktail. Then, 6 h after infection, groups IV and V, VI and VII, VIII and IX, X and XI, XII and XIII, and XIV and XV, were orally administered 0.5 ml of ISS, streptomycin (450 μg/ml), streptomycin (50 μg/ml), hibiscus acid (5 mg/ml), hibiscus acid (7 mg/ml) and hibiscus acid/streptomycin (5 mg/ml / 50 μg/ml) solutions, respectively. Each of the treatments with the test solutions and the ISS were administered to the mice every 12 h for 7 days. The presence of STEC^+^ and *S.* Typhimurium^+^ in the mouse faeces was quantified, and the mouse mortality rate and pathological manifestations were examined as reported by Portillo-Torres et al. (2022). Briefly, under aseptic conditions, faeces were collected from each cage bed every 8 h and stored under refrigeration. The faeces of the test animals were taken directly from the sawdust found in the base of each of the cages, containing each group of mice. The faeces were taken with sterilised forceps and placed in plastic bags with a hermetic closure. Every 24 h, the bags containing the faeces of each group of rodents were transported to the laboratory in refrigeration and under aseptic conditions. In the laboratory, the faeces of each 24 h-period were mixed and numbered for R^+^ pathogenic bacteria. The bacterial counts were determined for each of the 9 study groups. The sawdust from each of the 9 cages was changed and the cage was sterilised daily during the collection of the stool samples to avoid cross-contamination. To prepare for enumeration of EHEC R^+^ and *S*. Typhimurium R^+^ in each stool sample, 9 ml of a sterile peptone diluent (0.1%) was placed in the plastic bag containing 1.0 g of stool, then the faeces were homogenised manually by rubbing the bag from outside for 1 minute. The enumeration of the pathogenic R^+^ bacteria was performed by the pour plate technique using TSA supplemented with rifampicin (100 mg/l), and incubating at 35 ± 2 °C. Each dilution was inoculated in triplicate. To confirm the presence of the R^+^ mutant strain in the TSA-rif-plates, the colonies from these plates were taken and streaked onto eosin and methylene blue (EMB) agar or brilliant green agar (BGA), both containing rifampicin (100 mg/l) for EHEC R^+^ or *S*. Typhimurium R^+^, respectively.

The mortality rate of the mice in the different groups was calculated as the number of mice that died during the experiment, against all the mice used in each group. Throughout the study, the consistency of the faecal matter of each rodent was registered. The animals were also observed daily for any physiological and pathological abnormalities (weight loss, loss of appetite, weakness/slow movement and mortality) during the period of the experiment.

### Statistical analysis

The experiments for the MIC/MBC were repeated three times. An exploratory data analysis was performed to assess the assumptions of the equality of variances and normal distribution of errors of the results obtained from the *in vitro* antimicrobial activity of hibiscus acid and streptomycin, which were analysed using the Statgraphics Centurion XVI statistical program (StatPoint Technologies USA software, 2009) for the one-way analysis of variance. Comparisons of the means with the Tukey test were performed for each experimental section, with a significance level of *P* < 0.05.

## RESULTS AND DISCUSSION

### Minimum inhibitory concentration and minimum bactericidal concentration

The values obtained for the MIC and MBC of hibiscus acid, streptomycin, and the hibiscus acid/streptomycin against *S*. Typhimurium^+^ and STEC^+^ are reported in Table 1.

**Table 1:** Minimum inhibitory concentration (MIC), minimum bactericidal concentration (MBC), and fractional inhibitory concentration index (FICI) values of the hibiscus acid, streptomycin (STR), and hibiscus acid/streptomycin (HA/STR)

Bacteria cocktail	Treatments										FICI	interpretation
	hibiscus acid				streptomycin				HA/STR			
	MIC (mg/ml)	MBC (mg/ml)	MBC/MIC (mg/ml)		MIC (μg/ml)	MBC (μg/ml)	MBC/MIC (μg/ml)		MIC (mg/ml / μg/ml)	MBC (mg/ml / μg/ml)		
*S*. Typhimurium	7 ± 0.0	7 ± 0.0	1		300 ± 0.0	400 ± 0.0	1.8		3 ± 0.0/20 ± 0.0	5 ± 0.0/50 ± 0.0	0.488	synergism
STEC	7 ± 0.0	7 ± 0.0	1		300 ± 0.0	450 ± 0.0	1.8		3 ± 0.0/20 ± 0.0	5 ± 0.0/50 ± 0.0	0.488	synergism

STEC = Shiga-toxin-producing *Escherichia coli*

It should be noted that the MIC values that were obtained for the hibiscus acid were 7 mg/ml for both *S*. Typhimurium^+^ and STEC^+^, while for streptomycin, the MIC was 300 μg/ml for both pathogenic strains. However, when the mixture of both agents was tested, the MICs of the hibiscus acid/streptomycin for STEC^+^ and *S*. Typhimurium^+^ were 3 mg/ml and 20 μg/ml, respectively. A similar reduction was observed in the MBC values of the hibiscus acid and streptomycin, both alone and in mixture (Table 1).

In this study, the MIC and MBC for the STEC and *S*. Typhimurium strains resistant to multiple antibiotics were very high compared to the levels shown by strains sensitive to these antibiotics. This means that, to control an infection caused by these pathogenic strains in a human or animal, a very high level of streptomycin would be required, which would carry risk due to its toxicity. It is widely documented that streptomycin, even at the levels administered to control an infection by pathogenic bacterial strains not resistant to the antibiotic, presents a certain degree of toxicity (Peloquin et al. 2004).

### Fractional inhibitory concentration index (FICI)

The mixture of hibiscus acid with streptomycin gave an FICI of 0.488 for both *S*. Typhimurium and EHEC, showing a synergistic effect (Table 1). Synergistic and additive interactions between two antibacterial components have been reported to improve antibacterial efficacy compared to when they are used alone (van Gent et al. 2022).

### The antimicrobial effect in CD-1 mice infected with STEC or *S*. Typhimurium

The results of the antibacterial activity of the hibiscus acid, streptomycin, and the hibiscus acid/streptomycin mixture in the CD-1 mice infected with *S*. Typhimurium R^+^ or STEC R^+^ are reported in Tables 2 and 3. Both *S*. Typhimurium^+^ and STEC^+^ were able to colonise the mice and replicate in the mice that were treated with ISS-only; streptomycin 450 μg/ml; streptomycin 50 μg/ml; or hibiscus acid at 5 mg/ml (Table 2). By contrast, when the mice were administered with hibiscus acid 7 mg/ml or hibiscus acid/streptomycin 5 mg/ml / 50 μg/ml, both pathogens were no longer detected in the faeces after day one (Table 2). It is important to note that when hibiscus acid was tested at a concentration lower than the MBC (5 mg/ml) it had no effect on the survival of both pathogenic bacteria. However, when it was tested at the same concentration, but in a mixture with streptomycin, there was an effect in reducing the concentration of both pathogens and inactivating them. This confirms the synergistic effect previously observed in the culture broth.

**Table 2:** Effect of the treatments in the CD-1 mice on the faecal excretion of STEC^+^ and *S.* Typhimurium^+^ (CFU/g)

Groups	Treatments	Number of R^+^ bacteria excreted in the faeces of the mice each day throughout the study (CFU/g)							
		day 0	day 1	day 2	day 3	day 4	day 5	day 6	day 7
I (mouse)	ISS	0	0	0	0	0	0	0	0
II (mouse)	streptomycin	0	0	0	0	0	0	0	0
III (mouse)	hibiscus acid (7 mg/ml)	0	0	0	0	0	0	0	0
IV (mouse + *S*. Typhimurium^+^)	ISS	0	*5 × 10^2^	4 × 10^3^	7 × 10^3^	3 × 10^4^	8 × 10^4^	1 × 10^5^	1.3 × 10^6^
V (mouse + STEC^+^)	ISS	0	6 × 10^2^	2 × 10^3^	9 × 10^3^	3 × 10^4^	2 × 10^5^	6 × 10^5^	4.3 × 10^6^
VI (mouse + *S*. Typhimurium^+^)	streptomycin	0	5 × 10^2^	2 × 10^3^	7 × 10^3^	2 × 10^4^	7 × 10^4^	2 × 10^5^	1.2 × 10^6^
VII (mouse + STEC^+^)	streptomycin	0	6 × 10^2^	1 × 10^3^	6 × 10^3^	3 × 10^4^	8 × 10^4^	3 × 10^5^	2.0 × 10^6^
VIII (mouse + *S*. Typhimurium^+^)	streptomycin (50 μg/ml)	0	5 × 10^2^	2 × 10^3^	7 × 10^3^	2 × 10^4^	7 × 10^4^	2 × 10^5^	1.0 × 10^6^
IX (mouse + STEC^+^)	streptomycin (50 μg/ml)	0	6 × 10^2^	1 × 10^3^	6 × 10^3^	3 × 10^4^	8 × 10^4^	3 × 10^5^	1.5 × 10^6^
X (mouse + *S*. Typhimurium^+^)	hibiscus acid (5 mg/ml)	0	4 × 10^2^	2 × 10^3^	4 × 10^3^	5 × 10^4^	4 × 10^4^	1 × 10^5^	9.5 × 10^5^
XI (mouse + STEC^+^)	hibiscus acid (5 mg/ml)	0	7 × 10^2^	3 × 10^3^	5 × 10^3^	1 × 10^4^	3 × 10^4^	1 × 10^5^	2.4 × 10^6^
XII (mouse + *S*. Typhimurium^+^)	hibiscus acid (7 mg/ml)	0	6 × 10^2^	0	0	0	0	0	0
XIII (mouse + STEC^+^)	hibiscus acid (7 mg/ml)	0	1 × 10^2^	0	0	0	0	0	0
XIV (mouse + *S*. Typhimurium^+^)	HA/STR 5 mg/ml / 50 μg/ml	0	8 × 10^1^	0	0	0	0	0	0
XV (mouse + STEC^+^)	HA/STR 5 mg/ml / 50 μg/ml	0	9 × 10^1^	0	0	0	0	0	0

*Represents the average of the three-repeat counts of the same stool sample of each group collected every 24 hours

+ = rifampicin-resistant; HA/STR = hibiscus acid/streptomycin; ISS = isotonic saline solution; *S.* Typhimurium^+^ = *Salmonella* Typhimurium^+^; STEC^+^ = Shiga-toxin-producing *E. coli*^+^

On the other hand, the mice in the groups that were infected with STEC^+^ or *S*. Typhimurium^+^ and were administered after infection with SSI-only, streptomycin 450 μg/ml, streptomycin 50 μg/ml, or hibiscus acid 5 mg/ml, developed clinical signs and they all died during the study. It is important to mention that these mice began to die between the fifth and sixth day. In contrast, the mice that were infected with STEC^+^ or *S*. Typhimurium^+^, but were administered 7 mg/ml or hibiscus acid/streptomycin mixture (5 mg/ml / 50 μg/ml), only two mice from each group exhibited any symptoms in the first 2 days following infection, but gradually recovered after the administration of hibiscus acid or hibiscus acid/streptomycin mixture, and none of the mice in these groups died (Table 3). This shows that hibiscus acid at the concentration tested (minimum bactericidal dose) has an antimicrobial effect in animals infected by both pathogens. Also, the mixture of hibiscus acid and streptomycin showed an antimicrobial effect in the mice examined (Table 2). It should be noted that the concentration of hibiscus acid used in the mixture was lower than when it was tested in a pure form. This suggests that hibiscus acid potentiates the effect of streptomycin and that due to this interaction, a very low concentration of streptomycin is needed in a mixture with hibiscus acid to inactivate both pathogens, even when they are resistant to streptomycin alone. These results suggest that even though streptomycin at the doses in which it is generally administered causes toxicity to animals and humans, it could continue to be used at a lower and non-toxic dose if mixed with plant antimicrobials such as hibiscus acid.

**Table 3:** Clinical signs and mortality observed in the groups of mice infected and not infected with *S*.  Typhimurium^+^ and STEC^+^ during the experiment

Groups	Treatments	Clinical symptoms or mortality			
		loss of weight*	loss of appetite	body weakness/slow movement	mortality rate
I (mouse)	ISS	0/6	0/6	0/6	0/6
II (mouse)	streptomycin (450 μg/ml)	0/6	0/6	0/6	0/6
III (mouse)	hibiscus acid (7 mg/ml)	0/6	0/6	0/6	0/6
IV (mouse + *S*. Typhimurium^+^)	ISS	6/6	6/6	6/6	6/6
V (mouse + STEC^+^)	ISS	6/6	6/6	6/6	6/6
VI (mouse + *S*. Typhimurium^+^)	streptomycin (450 μg/ml)	6/6	6/6	6/6	6/6
VII (mouse + STEC^+^)	streptomycin (450 μg/ml)	6/6	6/6	6/6	6/6
VIII (mouse + *S*. Typhimurium^+^)	streptomycin (50 μg/ml)	6/6	6/6	6/6	6/6
IX (mouse + STEC^+^)	streptomycin (50 μg/ml)	6/6	6/6	6/6	6/6
X (mouse + *S*. Typhimurium^+^)	hibiscus acid (5 mg/ml)	6/6	6/6	6/6	5/6
XI (mouse + STEC^+^)	hibiscus acid (5 mg/ml)	6/6	6/6	6/6	5/6
XII (mouse + *S*. Typhimurium^+^)	hibiscus acid (7 mg/ml)	2/6	2/6	2/6	0/6
XIII (mouse + STEC^+^)	hibiscus acid (7 mg/ml)	2/6	2/6	2/6	0/6
XIV (mouse + *S*. Typhimurium^+^)	HA/STR 5 mg/ml / 50 μg/ml	2/6	2/6	2/6	0/6
XV (mouse + STEC^+^)	HA/STR 5 mg/ml / 50 μg/ml	2/6	2/6	2/6	0/6

*Number of affected mice/total number of mice in each group

+ = rifampicin-resistant; HA/STR = hibiscus acid/streptomycin; ISS = isotonic saline solution; *S*. Typhimurium^+^ = *Salmonella* Typhimurium^+^; STEC^+^ = Shiga-toxin-producing *E. coli*^+^

The absence of existing scientific studies published or readily available on the effects of hibiscus acid or *H. sabdariffa* extracts specifically on bacterial pathogens in livestock is a notable limitation. Despite the potential therapeutic properties attributed to hibiscus-derived compounds, particularly in the context of antibacterial activity, the literature lacks empirical evidence directly linking these compounds to livestock bacterial pathogens. Consequently, the interpretation of study results within the context of the available literature becomes challenging. Without established antecedents or prior investigations examining the effects of hibiscus acid on livestock bacterial pathogens, it is difficult to provide a substantive discussion or draw meaningful comparisons. Acknowledging this limitation underscores the necessity for future research endeavours to address this gap and conduct comprehensive investigations to elucidate the potential impact of hibiscus-derived compounds on bacterial pathogens in livestock settings.

We assessed the antibacterial efficacy of streptomycin, hibiscus acid, and their combination against multidrug-resistant STEC and *S.* Typhimurium using CD-1 mice. Notably, the combination demonstrated reduced MIC and MBC values compared to the individual compounds, indicating a potential synergistic effect. The observed pathogen elimination and increased survival rate in the treated mice signify a significant advancement in combating antibiotic-resistant infections. However, further studies are required.

An alternative approach for future research could involve elucidating the optimal dosing regimen and administration route for the hibiscus acid/streptomycin combination in livestock settings. This could entail conducting dose-response studies to determine the most effective concentrations of hibiscus acid and streptomycin for combating multidrug-resistant pathogens while minimising any potential adverse effects.

Additionally, exploring the pharmacokinetics and pharmacodynamics of the hibiscus acid/streptomycin combination in livestock would provide valuable insights into its absorption, distribution, metabolism, and excretion properties. This could involve studying the bioavailability and tissue distribution of both compounds individually and in combination, as well as assessing their potential for drug-drug interactions.

Furthermore, conducting field trials or longitudinal studies in livestock farms to evaluate the practicality and effectiveness of administering the hibiscus acid/streptomycin combination under real-world conditions would be invaluable. This could involve monitoring the incidence of bacterial infections, antibiotic resistance patterns, and livestock health outcomes over an extended period to assess the long-term efficacy and sustainability of this treatment approach.

To the best of our knowledge, this is one of the first reports in the literature of the antimicrobial effect of hibiscus acid, both alone and in a mixture with an antibiotic, in an animal model.
